# Post-operative Horner's Syndrome Following Total Thyroidectomy: A Case Report

**DOI:** 10.7759/cureus.27742

**Published:** 2022-08-07

**Authors:** Eleanor M Palmer, Prithvirao Sonoo, Imran Jawaid, Ahmed Javed

**Affiliations:** 1 Ophthalmology, Nottingham University Hospitals NHS Trust, Nottingham, GBR; 2 Medicine, Nottingham University Hospitals NHS Trust, Nottingham, GBR

**Keywords:** head and neck tumors, metastatic papillary thyroid cancer, thyroid cancer surgery, open thyroidectomy, thyroidectomy, post-thyroidectomy complication, sympathetic chain, horner’s syndrome

## Abstract

The oculosympathetic chain is a three-neuron pathway responsible for sympathetic innervation to the eye, which follows a complex anatomical course through the head and neck. Neck surgery may cause injury to this pathway, causing loss of sympathetic innervation producing the eponymous Horner’s syndrome (ipsilateral ptosis, miosis and anhidrosis), but this is rare in the reported literature. We present the case of a 23-year-old female who underwent total thyroidectomy for a right-sided, metastatic papillary thyroid carcinoma. Following surgery, in the immediate postoperative period, she was noted to have unilateral ptosis and miosis. This patient was assessed by an ophthalmologist due to persistent unilateral ocular symptoms following thyroidectomy. She was subsequently diagnosed with right-sided Horner’s syndrome. The diagnosis was confirmed following the observed reversal of her ocular symptoms using apraclonidine 1% minims. The management of Horner’s syndrome following thyroidectomy is conservative if no evidence of compressive hematoma or seroma is identified as in this case. The patient was followed up at six weeks following thyroidectomy and a partial improvement in ptosis was noted. The patient also reported blurred vision secondary to increased refractive error due to reduced pupillary function in her right eye. Prognosticating recovery from Horner’s syndrome following thyroidectomy is challenging due to limited evidence. Horner’s syndrome as a possible complication of thyroidectomy should be counselled to patients pre-operatively. A residual deficit from Horner’s syndrome may cause functional impairment in addition to the poor cosmetic outcome.

## Introduction

Sympathetic innervation to the eye follows a complex anatomical course. It originates in the hypothalamus, traverses the brainstem to reach the ciliospinal centre of Budge (C8-T2) in the spinal cord and then completes a full about-turn to reach the superior cervical ganglia. Post-ganglionic fibres then pass in close proximity to the internal carotid artery to their final effector muscles. These include the dilator pupillae muscle responsible for pupillary dilatation, and the Muller’s muscle, which is responsible for retraction of the upper eyelid. Three separate neurones are involved in the transmission of sympathetic innervation to these muscles, and injury at any point can result in the eponymous Horner’s syndrome (HS), classically defined as a triad of ipsilateral ptosis, miosis and anhidrosis. This may be accompanied by ipsilateral vascular dilatation of the facial blood vessels.

One possible mechanism of injury to the sympathetic innervation to the eye is through head and neck surgery. This is most commonly encountered in procedures requiring central neck dissection of the structures surrounding the sympathetic trunk such as thyroidectomy for malignant lesions of the thyroid. For most thyroid cancers, total thyroidectomy is preferred for optimal clearance, though regional lobectomy may be offered in patients whose tumour is confined to a small area, usually less than 1 cm [1]. Central neck dissection involving removal of local paratracheal lymph nodes is typically recommended alongside total surgical excision of the thyroid to decrease the likelihood of recurrence, particularly for papillary thyroid carcinomas in which lymphatic spread is common [1]. Other adjunctive therapies may include radioactive iodine therapy, chemotherapy, external beam radiotherapy and, more recently, the use of immunotherapy [2].

Here, we describe the case of a patient who underwent thyroidectomy for metastatic papillary thyroid carcinoma and experienced post-operative Horner's syndrome as an immediate complication of the procedure.

## Case presentation

A 23-year-old Caucasian female who underwent a total thyroidectomy was referred postoperatively to the ophthalmology department. She complained of a progressively worsening right-sided partial ptosis, headache and mild visual disturbance following surgery.

The patient had a primary diagnosis of right thyroid papillary carcinoma with right-sided metastatic cervical lymph node involvement for which she was offered total thyroidectomy as treatment. At the age of 23 years, she had originally presented to her general practitioner after noticing a unilateral, right-sided, non-tender neck mass and was subsequently referred onwards to Ear, Nose and Throat (ENT) services. She reports that prior to noticing the lesion, she suffered noticeable changes in her mood and sleep during the four years prior to this presentation. She was investigated with ultrasound, computerised tomography and histological analysis of core biopsies from the lesion. After a discussion of the results of the above investigations at the ENT multi-disciplinary team meeting, she was offered a total thyroidectomy with bilateral level VI neck dissection, with an additional level II-IV dissection of the right neck lymph nodes.

No intraoperative complications were recorded in the operative notes. Comments from the surgeon performing the procedure noted level VI disease encasing the right recurrent laryngeal nerve. Bilateral neck drains were inserted. On assessment in the anaesthetic recovery room, the surgeon noted right-sided incomplete ptosis and a right miosed pupil in the immediate postoperative period. The patient reported a subjective progressive worsening of her new onset ptosis in addition to a new onset occipital headache. She did not report any ipsilateral anhidrosis, nor any voice changes or swallowing difficulties. No evidence of neck haematoma or seroma was observed after careful clinical assessment. She was assessed by an ophthalmologist (AJ) on day four postoperatively, who assessed her case given the context of her recent procedure. She had no previous ocular history and no relevant past medical history. She wore corrective glasses for hypermetropia. On assessment, her visual acuity in both eyes was +0.0 logMAR unaided (6/6 Snellen). All cranial nerves were intact on assessment.

The following was observed on examination using slit-lamp biomicroscopy (Table 1). The examination was limited due to positioning difficulties.

**Table 1:** Examination findings on ophthalmic assessment using slit-lamp biomicroscopy (NB: Normal intraocular pressure: 10-21 mmHg)

	Right eye	Left eye
Lids/external	Partial ptosis	No abnormalities
Cornea	Clear	Clear
Anterior chamber	Deep and quiescent	Deep and quiescent
Pupil appearance (undilated), Photopic conditions, Scotopic conditions	Regular - No relative afferent pupillary defect, Miosed (2 mm), Miosed (2 mm)	Regular - No relative afferent pupillary defect, Normal (3 mm), Normal (4 mm)
Intraocular pressure	14 mmHg	16 mmHg
Fundus	No abnormalities	No abnormalities

Investigations

**Figure 1:**
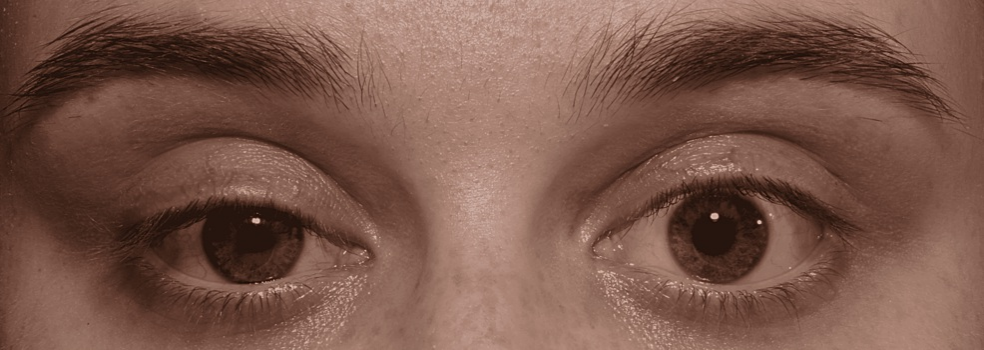
Clinical image taken before bilateral apraclonidine 1% instillation

**Figure 2:**
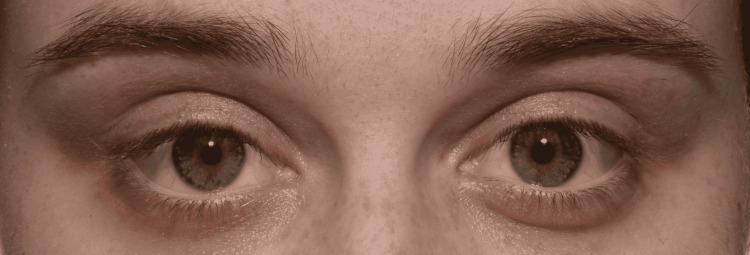
Clinical image taken one hour after bilateral apraclonidine 1% instillation

Pupil size was recorded prior to instillation of apraclonidine 1% eye drops to both eyes. After a period of 60 minutes, the patient was re-examined and the pupil size recording was repeated. Subjective improvement in the appearance of the ptosis was noted by the patient and observed clinically thus confirming the diagnosis of HS (Table 2) (Figures 1-2).

**Table 2:** Change in pupil size recorded before and following bilateral apraclonidine 1% instillation

	Right pupil	Left pupil
Prior to apraclonidine 1% instillation	2 mm	3 mm
60 minutes after apraclonidine 1% instillation	3 mm	3 mm

Treatment

Conservative management was offered following assessment by both ophthalmology and ENT services, as no further surgical intervention was indicated. The patient struggled with the cosmetic deficit caused by the residual ptosis. She was therefore offered apraclonidine 1% eye drops to take home on discharge to use on a pro re nata basis as needed.

Outcome

The patient was reviewed post-operatively as an outpatient under the ENT department via a telephone appointment service for review of repeat serum calcium levels. A few weeks following discharge, she attended her local optician after noticing blurred vision and headaches. The optician noted on refraction that her prescription had become increasingly hypermetropic and was corrected using spectacles. She was also subsequently diagnosed with visual migraines. At six weeks postoperatively, she reported a mild improvement in her right-sided ptosis, though complained of a persistently miosed right pupil. She also reported unilateral muscle weakness in her right arm, which had developed following the procedure for which she was receiving community physiotherapy. She continues to be followed up under the ENT service and oncology clinic.

The patient reported a significant negative psychological impact from the residual HS. She had tried to search online about HS as a complication following thyroidectomy but struggled to find readily accessible information. Pre-operatively, the possibility of HS as a complication of thyroid surgery had not been discussed. She was able to communicate with other patients via social media who had also suffered from HS as a complication of neck surgery and similarly encountered difficulties in obtaining information about the condition and its prognosis.

## Discussion

First described in 1869 by Horner [3] as a syndrome of ipsilateral ptosis, miosis and facial anhidrosis, HS may be caused by a lesion to any of the three sympathetic neurons that innervate the eye. It therefore must be investigated within the context of the patient’s presentation, for example, as was noted in this report as an immediate postoperative complication of neck surgery. HS was first reported as a complication following thyroidectomy in 1915 and was particularly associated with postoperative wound infection [4]. Over the last 10 years, 18 cases of post-thyroidectomy HS have been reported in the scientific database PUBMED/MEDLINE, though the true incidence is likely higher. Overall, it is thought to be a relatively rare complication, with one large centre reporting the rate of HS post-thyroidectomy at 0.2% in a cohort of almost 500 patients [5].

Thyroidectomy involves the excision of the thyroid gland and is commonly indicated for malignant disease. Neuronal injury may be produced by a number of different mechanisms, including direct damage to the stellate ganglion, use of lateral retractors and postoperative haematoma or nerve ischaemia if vascular supply is compromised [5-6]. Dissection of surrounding structures within the central neck compartment may also cause neuronal injury. A systematic review investigating the rate of HS following anterior cervical disc fusion neck surgery found that, typically, HS was observed as a hyperacute complication in the immediate postoperative period, which is comparable to findings observed in other reported cases of post-thyroidectomy HS [6-8]. Within the context of thyroid cancers, central neck dissection is utilised to reduce the risk of recurrent disease from lymphatic spread. Papillary thyroid carcinoma, the most common thyroid malignancy, frequently metastasises to local nodes and therefore, lymph node dissection is often indicated, either for prophylactic purposes or when metastatic disease is evident from pre-operative analysis as was the case for our patient [9]. However, despite the accepted convention of performing central neck dissection for lymph node clearance in proven metastatic disease, some studies question the use of central neck dissection for prophylactic purposes due to the lack of efficacy in preventing recurrent disease [10-11] and thus the issue of prophylactic lymph node clearance remains controversial. This is reflected in the varying consensuses on indications for central neck dissection from international advising surgical and oncological bodies [12-14].

Though the characteristic triad of features (ipsilateral ptosis, miosis and facial anhidrosis) may be observed in HS, facial anhidrosis is not typically observed as a feature of post-thyroidectomy HS in other reported cases [6,8] and similarly was not observed in our patient. Investigation using apraclonidine eye drops to confirm the diagnosis of HS is a useful tool [15]. Apraclonidine is available in either 0.5% or 1% concentrations. Diagnosis relies on the principle of denervation supersensitivity following an injury to the sympathetic neuronal pathway, where neurons downstream of the lesion are artificially stimulated using an alpha-adrenergic agonist, causing a reversal of ptosis and miosis. The advantage of using apraclonidine as a diagnostic tool is its easy accessibility, relative inexpensiveness, minimal side effects and high diagnostic yield after only 45 minutes following instillation [16]. As a diagnostic test, one report estimates its sensitivity at 93% for the diagnosis of HS of any cause [15]. It prevents the unnecessary use of CT or MRI imaging, which, though of clinical value for patients with HS of unknown aetiology, such as carotid artery dissection, stroke or apical lung tumours, pose additional risks, such as radiation exposure, or are contraindicated as in the case of patients with previous metallic prostheses or implants. The limitation of apraclonidine as a diagnostic test is the possible false negative result, which may be produced in some cases of HS where denervation supersensitivity takes longer to develop following a neuronal injury. It should also be avoided in infants due to the risk of bradycardia and hypertension [16].

Treatment of HS is variable due to the wide range of possible underlying causes. In the case of post-thyroidectomy HS, treatment is often conservative. Prognosis of neuronal recovery is difficult to estimate, as some patients experience full recovery [5], but case report evidence suggests that the majority experience persistent deficit [4,6-7,17]. Though this may be long-term, the impact of HS is predominantly cosmetic and does not typically affect visual function. One study suggests the use of short-term neurotrophic therapy, such as vitamin B1 and vitamin B12, to encourage neuronal repair [18]. The psychological patient impact of persistent HS has not been studied to date. For our patient, the cosmetic deficit from HS was the most concerning and psychologically distressing aspect of her postoperative recovery. Qualitative analysis of future cases of post-thyroidectomy HS cases is warranted to aide such patients in coping with persistent oculomotor defects. For a select patient group for whom the cosmetic deficit from HS is not tolerated, apraclonidine drops can be prescribed as a short-term option, though duration and frequency should be monitored due to potential dose-dependent tissue toxicity and wider systemic effects [19].

## Conclusions

In conclusion, HS describes an injury to the oculosympathetic neuronal pathway, which may be produced by a variety of mechanisms and is a rare complication following total thyroidectomy for thyroid carcinoma, particularly when performed in conjunction with central neck dissection for lymph node clearance. The indication for central neck dissection is controversial due to contrasting evidence on recurrence rates, and the practice varies between centres. Diagnosis of HS is usually made in the immediate postoperative period as a hyperacute manifestation of neuronal injury and can be confirmed easily using apraclonidine eye drops. Treatment is usually conservative and the time to recovery variable. Patients undergoing thyroidectomy with residual HS can struggle with the cosmetic deficit caused by neuronal paralysis and should therefore be counselled pre-operatively regarding the risk of this, particularly in cases where there is extensive metastatic disease surrounding key pupillomotor sympathetic structures and injury, therefore, more likely to occur.
